# Effect of Differently Polarized Human Macrophages on the SH-SY5Y Cells Damaged by Ischemia and Hypoxia *In Vitro*

**DOI:** 10.1155/2023/5595949

**Published:** 2023-08-31

**Authors:** Ivan Mikhailovich Rashchupkin, Ekaterina Yakovlevna Shevela, Aleksandra Aleksandrovna Maksimova, Marina Aleksandrovna Tikhonova, Aleksandr Anatolevich Ostanin, Elena Removna Chernykh

**Affiliations:** Scientific Research Institute of Fundamental and Clinical Immunology, Novosibirsk, Russia

## Abstract

Macrophages are the major cells of innate immunity with a wide range of biological effects due to their great plasticity and heterogeneity. Macrophages play a key role in neuroregeneration following nervous tissue injury. However, the neuroregenerative potential of various macrophage phenotypes, including those polarized by efferocytosis, remains unexplored. The aim of this study was to compare the neuroregenerative and neuroprotective activity of soluble factors secreted by variously activated human macrophages on the functions of neural progenitors in an *in vitro* model of ischemia or ischemia/hypoxia. Macrophages were polarized by interferon-*γ* (M1), IL-4 (M2a), or interaction with apoptotic cells (M2(LS)). The effect of macrophages conditioned media on the proliferation, differentiation, and survival of SH-SY5Y cells damaged by serum deprivation alone (ischemic conditions) or in combination with CoCl_2_ (ischemic/hypoxic conditions) was assessed. All studied macrophages stimulated the proliferation and differentiation of SH-SY5Y cells. On day 3, the pro-proliferating effect of M1 and M2 was similar and did not depend on the severity of the damaging effect (ischemia or ischemia/hypoxia), while on day 7 and under ischemic/hypoxic conditions, the effects of M2(LS) exceeded those of M1 and M2a cells. The prodifferentiation effects of macrophages were manifested in both short- and long-term cultures, mainly under ischemic/hypoxic conditions, and were most characteristic of M2(LS) cells. Importantly, the ischemia/hypoxia model was accompanied by the pronounced death of SH-SY5Y cells. Only macrophages with the M2 phenotype demonstrated antiapoptotic activity, and the effect of M2(LS) was higher than that of M2a. The results obtained indicate that human macrophages have neuroprotective and neuroregenerative activity, which is mediated by soluble factors, is most characteristic for macrophages activated by efferocytosis (M2(LS)), and is most prominent under *in vitro* conditions simulating the combined effect of ischemia/hypoxia.

## 1. Introduction

At present, there is no doubt that the adult nervous system, due to its plasticity, is able to regenerate [1]. The concept of neuroregeneration comprises both the recovery of damaged nerve cells (intracellular regeneration) and the formation of new cells (cellular regeneration). The former case involves regeneration and remyelination of neurites, synaptogenesis, and restoration of damaged cell ultrastructures, and the latter case is related to the formation of new neurons from neural stem cells (NSCs) [2]. Replacement of damaged neurons with new cells requires enhancement of NSC proliferation, migration of NSCs to the damaged tissue, and their subsequent differentiation with integration into the existing neural network. These processes are regulated by immune system cells, in particular by macrophages that are represented in the central nervous system by microglia and recruited monocytic macrophages, with the latter playing an important role in pathological conditions.

Macrophages, which are characterized by pronounced plasticity, are able to alter their functional phenotype in response to various microenvironment signals and form many transitional phenotypes between opposite activation states: classical M1 and alternative M2 [3]. M1 phenotype is characterized by proinflammatory activity, whereas M2, on the contrary, exhibits anti-inflammatory properties, provides resolution of inflammation, and stimulates reparative processes [4]. In pathological conditions, the M1/M2 balance is drastically shifted toward the M1 phenotype that mediates neuroinflammation. Restoring M1/M2 balance and resolution of inflammation trigger neuroregeneration [5]. In this case, the intensity and coordination of these processes determine the degree and speed of impaired functional recovery.

According to the literature, the classical activation of macrophages by lipopolysaccharide and interferon-gamma (IFN-*γ*) induces the M1 phenotype, whereas the alternative activation by IL-4 and IL-13 induces the M2a phenotype. In addition, the M2 phenotype can be induced by other factors: immune complexes (M2b), IL-10, and dexamethasone (M2c) [3]. One of the most important inducers of the M2 phenotype is the uptake of apoptotic cells (efferocytosis) [6]. Previously, we developed an original protocol for the generation of M2-like macrophages (low serum, M2(LS)), which is based on culturing monocytes undergrowth/serum factor deprivation, which promotes deprivation apoptosis and subsequent uptake of apoptotic cells by macrophages [7]. Macrophages generated in this way are characterized by reduced allostimulatory activity, low production of proinflammatory cytokines, and high production of growth and neurotrophic factors (vascular endothelial growth factor (VEGF), erythropoietin (EPO), brain-derived neurotrophic factor (BDNF), basic fibroblast growth factor, epidermal growth factor (EGF)) [7, 8].

Although the role of M1 and M2 macrophages/microglia in inducing and resolving inflammation has been characterized quite fully, their involvement in the regulation of neurogenesis has not been well studied, and there are no data on efferocytosis-induced M2 cells. Therefore, the aim of this study was to comparatively assess the effect of human macrophages with M1, M2a, and M2(LS) phenotypes on the proliferative activity, viability, and differentiation of NSCs under simulated ischemic and combined ischemic and hypoxic conditions *in vitro*.

The SH-SY5Y neuroblastoma cell line was chosen as an *in vitro* model of human NSCs. The cells of this line exhibit properties of neural progenitors: they express NSC markers (nestin, doublecortin), actively proliferate, tend to form clusters and neurospheres [9], and are capable of differentiating into dopaminergic and cholinergic neuron-like cells under the action of various stimuli [10]. *In vitro* ischemic conditions were simulated by partial deprivation of growth/serum factors. Hypoxia was induced by the chemical hypoxia inducer CoCl_2_, which is a hypoxia-induced factor stabilizer [11].

## 2. Results

### 2.1. Characterization of Macrophages

**Figure 1:**
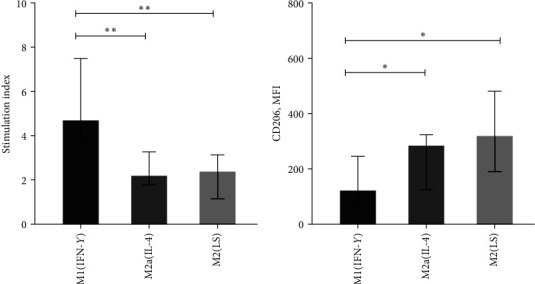
Characteristic of M1(IFN-*γ*), M2a(IL-4), and M2(LS) macrophages: (a) allostimulatory activity, (b) expression of CD206.  ^*∗*^*p* < 0.05,  ^*∗∗*^*p* < 0.01 (the Mann–Whitney *U* test). MFI, mean fluorescence intensity. Data are expressed as the median and interquartile range (Me (IQR, 25%–75% quartiles)). (a) *n* = 13, (b) *n* = 8.

First of all, we evaluated the capacity of M1(IFN-*γ*), M2a(IL-4), and M2(LS) macrophages to stimulate T-cell proliferation in mixed leukocyte culture, or allostimulatory activity and the expression of the M2-associated marker (CD206) by macrophages. Allostimulatory activity is an integral parameter that reflects a number of macrophage characteristics (human leukocyte antigen-DR isotype expression, coinhibitory and costimulatory molecules expression, cytokine production, capacity to induce regulatory T cells, etc.) and allows identification of M1 and M2 macrophages [12]. The studied macrophages significantly differed from each other in allostimulatory activity. Thus, M1 macrophages polarized by IFN-*γ* were characterized by higher allostimulatory activity compared to M2a(IL-4) and M2(LS) (*p* < 0.05, Figure 1(a)). We also studied the level of CD206 expression, which is considered a characteristic marker of M2 macrophages. As expected, M2a(IL-4) and M2(LS) expressed CD206 at a higher level than M1 (*p* < 0.05, Figure 1(b)). Thus, M1(IFN-*γ*), M2a(IL-4), and M2(LS) macrophages corresponded to the characteristics typical for their phenotype.

### 2.2. Effect of Soluble Factors of Human Macrophages on SH-SY5Y Cell Proliferation, Viability, and Yield in an Ischemic In Vitro Model

**Figure 2:**
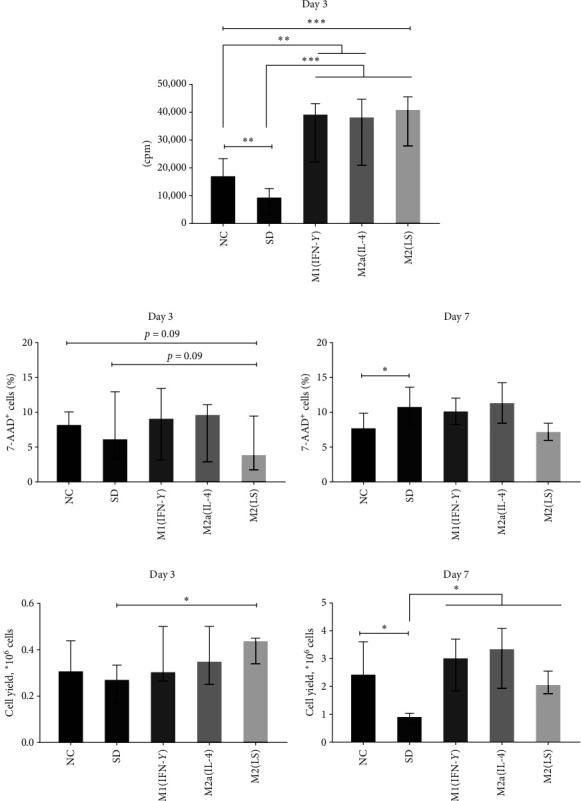
Effect of conditioned media from differently activated macrophages on proliferative activity (a), survival (b), and the cell yield (c) of SH-SY5Y cells under serum deprivation.  ^*∗*^*p* < 0.05,  ^*∗∗*^*p* < 0.01,  ^*∗∗∗*^*p* < 0.001 (the Mann–Whitney *U* test and *t*-test). cpm, counts per minute (proliferation was assessed using radioisotope method); 7-AAD, 7-aminoactinomycin D, NC, normal conditions; SD, serum deprivation conditions. Data are expressed as median and interquartile range (Me (IQR, 25%–75% quartiles)). (a) *n* = 9, (b) *n* = 4–8, (c) *n* = 4–12.

Under normal conditions (NC, 10% fetal bovine serum (FBS)), SH-SY5Y cells actively proliferated and reached a monolayer in 3–4 days. An analysis of the proliferative response kinetics showed a gradual increase in the proliferative activity of cells, which reached its maximum after 72 hr cultivation (data not shown). Under ischemia-like conditions (partial serum deprivation, *SD*, 1% FBS), SH-SY5Y cells also proliferated, but the intensity of their proliferation after 72 hr was significantly reduced compared with that in standard cultures (*p* < 0.05) (Figure 2(a)). Conditioned media (30% v/v), which were derived from classically activated M1(IFN-*γ*), alternatively activated M2a (IL-4), and M2(LS) macrophages polarized through efferocytosis, exerted a comparable stimulating effect and increased cell proliferation 4.2–4.5-fold.

Since enhanced cell proliferation could be connected with increased survival due to a decrease in SH-SY5Y cell death, we next evaluated the number of nonviable cells (7-AAD^+^) in SH-SY5Y cell cultures supplemented with M1, M2a, and M2(LS) macrophage conditioned media (CM) (Figure 2(b)). SD in both 3-day and 7-day cultures did not lead to an increase in the percentage of nonviable cells. Neither M1 CM nor M2 CM significantly changed the number of dead cells. We observed only a slight decrease (*p*=0.09) in nonviable cells (compared with both normal conditions and deprivation control) on day 3 in the presence of M2(LS) CM.

In addition, we also evaluated the effect of macrophage CM on the number of SH-SY5Y cells since cell yield may reflect both proliferative activity and the level of cell apoptosis. Compared with NC, SD decreased the cell yield 1.2-fold on day 3 (*p* > 0.05) and more than twofold on day 7 (*p* < 0.05). The addition of CM from all macrophage types caused a 1.4–1.6-fold increase in the number of cells compared with that in deprivation controls as early as on day 3, which was statistically significant only for M2(LS) (Figure 2(c)). At day 7, CM derived from all studied macrophages caused a more than twofold increase in the cell yield compared with that in deprivation controls (*p* < 0.05). Furthermore, M1, M2a, and M2(LS) CM did not differ significantly in the level of stimulating effect. Thus, M1, M2a, and M2(LS) soluble factors enhanced the growth of SH-SY5Y cells under simulated ischemic conditions *in vitro*, and this effect was observed regardless of the macrophage phenotype.

### 2.3. Effect of Soluble Factors of Human Macrophages on SH-SY5Y Cell Differentiation in an Ischemic In Vitro Model

**Figure 3:**
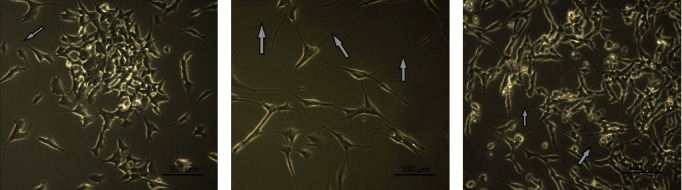
Morphological characteristic of intact and differentiated SH-SY5Y cells: (a) intact cells, (b) RA-differentiated cells, (c) cells cultivated under serum deprivation conditions (1% FBS) in the presence of M2(LS) conditioned media. Arrows indicate long neurites. RA, retinoic acid; FBS, fetal bovine serum. Day 7.

Because successful neuroregeneration requires not only proliferation but also neural progenitor differentiation, we evaluated the effect of macrophage CM on the differentiation of SH-SY5Y cells. Retinoic acid (RA) is a classic inducer of SH-SY5Y cell differentiation. During differentiation, cell bodies are elongated, and neurites are significantly elongated; at the later stages of differentiation, cells form synaptic-like connections with each other (Figure 3).

One of the main morphological criteria for differentiation is the predominance of the neurite length over the cell body length. Another criterion for cell differentiation is the average neurite length [13]. We evaluated the number of differentiated cells and the average neurite length at the early (day 3) and late (day 7) stages. As expected, the number of differentiated cells in the presence of RA (positive differentiation control) increased significantly on day 3 and continued increasing till day 7. The average length of cell neurites in RA-induced cultures was also significantly higher than that in deprivation controls at both time points (*p* < 0.05) (Table 1).

Similar to RA, although to a lesser extent, all studied macrophage CM increased the number of differentiated cells in comparison with deprivation controls on day 3 with a statistically significant effect only for M2(LS) (*p* < 0.05). Of note, the stimulatory effect of M2(LS) was more than twofold higher compared with that of M1 and M2a. In addition, M2(LS) enhanced the length of cell neurites more effectively than M1 and M2a (*p* < 0.05) (Table 1).

Cell culture prolongation up to 7 days did not result in a further increase in the relative number of differentiated cells and the average neurite length, which may be explained by active cell proliferation under these conditions. Similar to 3-day cultures, only M2(LS) had a significant stimulatory effect on SH-SY5Y cell differentiation. Thus, the prodifferentiation effects of M2(LS) exceeded the effects of M1 and M2a under SD.

### 2.4. Effect of Soluble Factors of Human Macrophages on SH-SY5Y Cell Proliferation in an Ischemic/Hypoxic In Vitro Model

**Figure 4:**
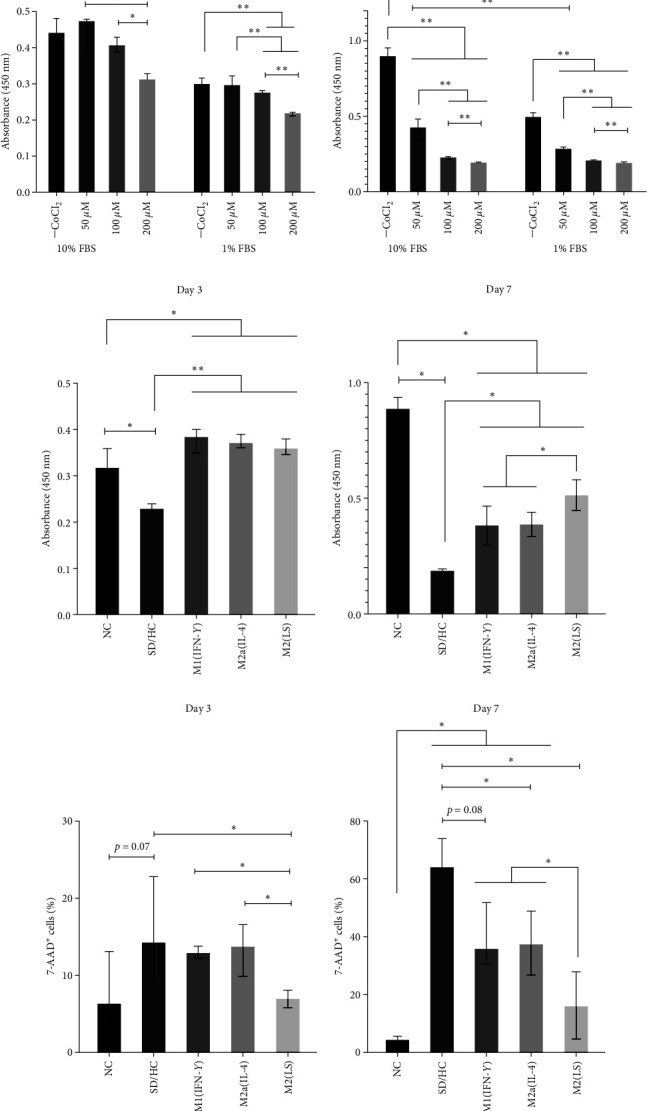
Effect of conditioned media from differently activated macrophages on proliferative activity and viability of SH-SY5Y cells under serum deprivation and hypoxia conditions: (a) proliferative response of SH-SY5Y cells in the presence of various doses of CoCl_2_ under normal (10% FBS) and serum deprivation (1% FBS) conditions; (b) effect of macrophage CM on SH-SY5Y proliferation; (c) effect of macrophage CM on SH-SY5Y viability. Percentage of 7-AAD^+^ cells among all SH-SY5Y cells is shown.  ^*∗*^*p* < 0.05,  ^*∗∗*^*p* < 0.01 (the Mann–Whitney *U* test and *t*-test). NC, normal conditions, SD/HC, serum deprivation, and hypoxia conditions; FBS, fetal bovine serum. Data are expressed as the median and interquartile range (Me (IQR, 25%–75% quartiles)). (a) *n* = 5, (Bb) *n* = 8, (c) *n* = 4–6.

As has already been shown, SD (1% FBS) decreased SH-SY5Y cell proliferation (1.3-fold) compared with that under NC (10% FBS) on day 3. The addition of CoCl_2_ to simulate the combined action of serum deprivation and hypoxia (SD/HC: serum deprivation/hypoxic conditions) at a concentration of 50 *μ*M did not enhance the inhibitory effect on proliferation. However, an increase in CoCl_2_ concentration (100 and 200 *μ*M) was associated with a dose-dependent decrease in proliferation. On day 7, cell proliferation under SD/HC was significantly reduced compared with that under NC in a dose-dependent manner (Figure 4(a)). Therefore, the SD/HC conditions demonstrated a more pronounced inhibitory effect on SH-SY5Y cell proliferation. Based on dose-dependence data, a concentration of 100 *µ*M CoCl_2_ was chosen for further experiments.

At the next stage, we evaluated the effect of CM derived from M1(IFN-*γ*), M2a (IL-4), and M2(LS) on SH-SY5Y cell proliferation under SD/HC (Figure 4(b)). Compared with NC, the proliferation of SH-SY5Y cells under SD/HC decreased 1.4-fold on day 3 (*p* < 0.05) and 4.8-fold on day 7 (*p* < 0.01), i.e., the cells almost ceased to proliferate. Macrophage CM significantly increased the level of proliferation both in 3-day and 7-day cultures. In 3-day cultures, the effects of CM of all studied macrophages were comparable and ranged from 1.6 to 1.65 (*p* < 0.05). In 7-day cultures, the stimulating effect of macrophage CM was also retained (*p* < 0.05). However, the effect of M2(LS) CM was higher compared with M1 CM and M2a CM (*p* < 0.05). Thus, soluble factors of all studied macrophages enhanced the proliferation of SH-SY5Y cells under SD/HC, with M2(LS) exerting a significantly more pronounced effect compared with that of M1 and M2a with longer exposure to damaging factors.

### 2.5. Effect of Soluble Factors of Human Macrophages on SH-SY5Y Cell Viability in an Ischemic/Hypoxic In Vitro Model

Further, we evaluated the neuroprotective effect of M1(IFN-*γ*), M2a (IL-4), and M2(LS) soluble factors, namely, the effect of CM on the viability of SH-SY5Y cells under SD/HC. Under SD/HC, the median number of nonviable SH-SY5Y cells increased by day 3 and amounted to 14.2% (9.6; 22.8) (median and (Q1; Q3 values)) versus 6.3% (3.0; 13.1) under NC (*p*=0.07; Figure 4(c)). At this stage, the protective effect was observed only for M2(LS) CM, which decreased the number of nonviable cells to 6.9% (5.7; 8.0) (*p* < 0.05). However, the viability of SH-SY5Y cells added with M1 CM (12.9% (12.2; 13.8) 7-AAD^+^ cells) and M2a CM (13.7% (9.8; 16.5)) did not differ from that in controls (*p* > 0.05).

Longer cultivation under SD/HC (7 days) led to a further increase in the percentage of nonviable cells: up to 64.0% (52.7; 73.8) versus 4.3% (3.1; 5.5) under NC (*p* < 0.05; Figure 4(c)). Only the M2 phenotype of macrophages exhibited a significant protective effect in these cultures, while M1 CM reduced the death of SH-SY5Y cells only in the form of a trend (35.6% (30.6; 51.8), *p*=0.08 compared with SD/HC). It is noteworthy that the protective effect of M2(LS) CM was significantly higher compared with that of M2a CM: the percentage of nonviable cells decreased to 37.2% (26.8; 48.9) for M2a CM and to 16.0% (4.7; 27.8) for M2(LS) CM. Thus, under combined SD/HC, soluble factors of M2 macrophages enhance the survival of SH-SY5Y cells. In this case, the protective effect of M2(LS) is observed at both early and late stages of cultivation and exceeds the similar effect of M2a cells.

### 2.6. Effect of Soluble Factors of Human Macrophages on SH-SY5Y Cell Differentiation in an Ischemic/Hypoxic In Vitro Model

Previously, we have shown that under SD, macrophage-derived soluble factors are capable of stimulating not only proliferation but also differentiation of SH-SY5Y cells, with M2(LS) exhibiting a significantly more pronounced prodifferentiation effect. Next, we evaluated whether a similar effect of macrophage CM persisted under SD/HC (i.e., in more pronounced injury). On day 3, the relative number of differentiated cells increased in the presence of CM from all studied macrophage phenotypes (Table 2). However, the percentage of differentiated cells in cultures with M2(LS) was 2.1–2.6-fold greater than that in cultures with M1 and M2a CM. In addition, an increase in the average neurite length was observed in the presence of macrophage CM, with all three studied macrophage phenotypes exerting a comparable effect (*p* < 0.05). Similar effects were seen in 7-day cultures, with the highest number and average neurite length being observed in M2(LS) cultures (Table 2).

Therefore, prodifferentiation effects of macrophage CM on SH-SY5Y cells observed under serum deprivation persisted in an SD/HC when damaging effects were enhanced. Importantly, M2(LS) CM had the highest stimulatory effect.

## 3. Discussion

The activation of neurogenesis has been considered an important mechanism of regenerative macrophage activity [14]. However, the efficiency of neurogenesis and its regulation by macrophages in neuropathology may be significantly affected by ischemia and hypoxia. Therefore, studying the effect of macrophages on NSCs under simulated ischemic or combined ischemic and hypoxic conditions seems to be an important aspect for a deeper understanding of the immune control of neuroregeneration and the development of new therapies for the treatment of neuropathology.

In the current study, we have studied the effects of soluble factors derived from differently activated human macrophages (M1(IFN-*γ*), M2a(IL-4), and M2(LS)) on proliferation, differentiation, and survival of SH-SY5Y cells under serum deprivation (an *in vitro* ischemia model) and a combination of deprivation and CoCl_2_-induced hypoxia (an *in vitro* ischemia/hypoxia model). According to the data obtained, both M1 and M2 macrophages are able to stimulate the proliferation and differentiation of SH-SY5Y cells. In 3-day cultures, the pro-proliferative effects of differently activated macrophages were similar and did not depend on the severity of the damaging effect (SD or SD/HC), while in 7-day cultures and under combined SD/HC, M2(LS) effects exceeded those of M1 and M2a cells. The prodifferentiation effects of macrophages were manifested both in short- and long-term cultures, mainly under SD/HC, and were most characteristic of M2(LS) cells. The neuroprotective effect of macrophage CM was revealed only for macrophages with the M2 phenotype, with the effect of M2(LS) significantly exceeding that of M2a macrophages. Altogether, neuroprotective and neuroregenerative activity is most characteristic of macrophages activated by efferocytosis and is associated with more pronounced damage to SH-SY5Y cells.

Despite M1 and M2 macrophages are capable of secreting pro- and anti-inflammatory cytokines and trophic and growth factors [7], each functional macrophage phenotype has a unique secretory profile [15]. For example, M2 cells are characterized by lower production of pro-inflammatory cytokines and higher production of trophic and growth factors [16]. The similar stimulating effects of soluble factors from all three macrophage types on the proliferation of SH-SY5Y cells may be explained by relatively low concentrations of the factors that are required to activate SH-SY5Y cells.

Data concerning the neuroregenerative capacity of microglia, i.e., tissue-specific macrophages, are restricted mainly to experimental animals and are often contradictory. For example, Vay et al. [17] showed that regardless of their polarization (with LPS or IL-4), rat microglia accelerated the differentiation of NSC into neurons while inhibiting NSC proliferation *in vitro*. On the contrary, according to the results by Osman et al. [18], neither NSC proliferation nor neuronal differentiation changed upon exposure to CM from LPS- or IL-4-stimulated mouse BV2 microglial cells.

Despite the fact that macrophages can engraft into the brain and replenish the functions of damaged microglia, there are only a few data on the neuroregenerative activity of monocyte-derived macrophages. As an example, Zhang et al. [19] demonstrated that CM from M2 but not M1 macrophages induced differentiation of NSC into neurons *in vitro*. In this study, the authors utilized macrophages obtained from mouse bone marrow and activated with M-CSF. In contrast, we first reported the stimulatory effect of human macrophages generated from blood monocytes in the presence of granulocyte macrophage colony–stimulating factor (GM-CSF), i.e., a factor simulating inflammatory conditions *in vitro*. Our results clearly demonstrated that not only M2 but also M1 macrophages are able to exhibit neuroprotective activity and enhance the proliferation/differentiation of neural progenitors. But as the damaging effect increases (e.g., combined ischemia and hypoxia), the neuroprotective effects of macrophages with the M2 phenotype exceed those of M1 cells. In addition, we obtained novel data showing that macrophages polarized by efferocytosis (M2(LS)) possess the most potent pro-regenerative activity.

Since we used macrophage-conditioned media, the described effects may be mediated by soluble factors. Notably, we characterized 47 cytokines in the supernatants of GM-CSF-differentiated macrophages polarized by efferocytosis, [8, 20] many of which (e.g., insulin-like growth factor 1 (IGF-1), VEGF, migration inhibitory factor (MIF), BDNF, EPO, EGF, TGF*α*, etc.) may contribute to the neuroprotective and neuroregenerative effect of macrophages [21–23]. Consistent with the above observations, we propose that M2 macrophages can significantly enhance neuronal differentiation of NSC-like SH-SY5Y cells due to the secretion of IGF-1, BDNF, VEGF, EPO, MIF, and many others.

Indeed, IGF-1 plays a key role in mediating the neuroprotective effect of macrophages. According to our previous data, the level of IGF-1 production by M2(LS) is tenfold higher than that by M- and GM-CSF-induced nonpolarized macrophages [7]. IGF-1 is able to induce neural differentiation of dental pulp stem cells and stimulate differentiation of SH-SY5Y cells *in vitro* [24, 25]. We suggest that IGF-1 could significantly contribute to the more pronounced induction of neural differentiation demonstrated by M2(LS). In addition, IGF-1 is able to stimulate the proliferation of SH-SY5Y cells [26]. Moreover, IGF-1 is known to be a potent protective factor; in particular, it exerts a glycogen synthase kinase-3-beta (GSK3b)-mediated antiapoptotic effect on neurons [27]. Therefore, a high level of IGF-1 secretion may largely explain the protective effect of M2(LS).

Another important inducer of neurodifferentiation, which is involved in a number of protocols for SH-SY5Y cell differentiation, is BDNF [28, 29]. According to our previous data, M2(LS) actively produced BDNF [7].

The VEGF and EPO may also be factors that contribute to mediating the neuroregenerative effects of macrophages. VEGF is not only an angiogenic factor but also has neuroprotective properties, which have been demonstrated both *in vitro* and *in vivo* [30, 31]. In addition, VEGF is involved in the regulation of neurogenesis by stimulating the proliferation of neuronal progenitors [32]. EPO is currently considered to exert neuroprotective, neurotrophic, and antiapoptotic effects [23]. In addition, EPO is known to stimulate the proliferation of neural progenitors [33]. According to our data, the level of VEGF production by M2(LS) significantly exceeds that by M1 and M2a [7]; also, efferocytosis significantly increases EPO production [34]. Altogether, VEGF may significantly contribute to mediating the anti-apoptotic effect of M2(LS).

Along with neurotrophic and angiogenic factors, the macrophage MIF has been considered another factor possessing neuroregenerative activity. Actually, MIF was shown to increase the survival and proliferation of mouse NSCs [35]. In a pilot clinical trial, the increased serum MIF in patients with cerebrovascular diseases following intranasal therapy with M2(LS) CM was associated with a potent clinical response [20].

Given the wide range of cytokines and growth factors that exhibit neuroregenerative activity, the effects of macrophages observed in our study are apparently mediated not by one specific factor but rather by their combination.

## 4. Conclusions

The results of the present study show that human macrophages possess neuroprotective and neuroregenerative activity, which is mediated by soluble factors, is most characteristic for macrophages activated by efferocytosis (M2(LS)), and is most pronounced under *in vitro* conditions simulating the combined effects of ischemia and hypoxia. The data obtained allow us to consider M2 macrophages as a promising cellular platform for the development of new methods for the treatment of inflammatory and degenerative diseases of the nervous system.

## 5. Materials and Methods

### 5.1. Isolation and Generation of Macrophages

Human blood samples were obtained from healthy donors with informed consent. Peripheral blood mononuclear cells (PBMCs) were isolated from heparinized blood through density gradient centrifugation (Ficoll-Paque, Sigma-Aldrich, St. Louis, MO, USA). Then PBMCs were plated at 3–5 × 10^6^/ml in tissue culture dishes (TPP, Trasadingen, Switzerland) and cultivated in RPMI-1640 (Biolot, Saint-Petersburg, Russia) supplemented with 2 mM sodium pyruvate, 0.3 mg/ml L-glutamine, 0.05 mM 2-mercaptoethanol, 1% nonessential amino acids, 100 *µ*m/ml gentamicin, 50 ng/ml recombinant human GM-CSF, and 10% FBS (FBS was added only for M1 and M2a macrophages) (all reagents of Sigma-Aldrich, St. Louis, MO, USA). After 1 hr, the nonadherent cells were discarded, and adhesive cells were cultured at 37°C with 5% CO_2_ for 7 days. At day 5, the polarizing stimuli were added: 200 ME/ml IFN-*γ* (Sigma-Aldrich) for M1 macrophages and 20 ng/ml IL-4 (Sigma-Aldrich) for M2a. M2(LS) generated under serum deficiency conditions were cultivated in a similar culture medium supplemented with 2% of autoplasma and without FBS. The adhesion time for M2(LS) was increased to 18 hr. After 7 days of cultivation, the conditioned medium of macrophages was collected, centrifuged (1,500 rpm, 7 min), and then stored at −80°C. For use in experiments, the conditioned media were thawed, filtered using a 0.22 *μ*m pore size filter, and added to SH-SY5Y cell cultures at a concentration of 30% (v/v).

### 5.2. Cultivation of SH-SY5Y Cells

SH-SY5Y cells (American Type Culture Collection, #CRL-2266, Manassas, VA) were cultured in DMEM/F12 (Biolot) supplemented with 10% FBS (normal conditions, NC) in T-25 cell culture flasks (TPP). Cells were passaged when 70%–80% of the monolayer was reached and then plated at 0.5–1 × 10^6^/ml. The number of passages did not exceed 15.

### 5.3. Ischemia and Hypoxia Modeling

In all experiments, ischemia was modeled by the partial deprivation of growth/serum factors: the concentration of FBS was reduced from 10% to 1% (serum deprivation, SD). Hypoxia was simulated by adding 100 *µ*M of the chemical hypoxia inducer cobalt chloride(II) (Sigma-Aldrich) (hypoxic conditions, HC).

### 5.4. Allostimulatory Activity and Phenotypic Characterization of Macrophages

The allostimulatory activity of macrophages was determined by measuring allogeneic T-cell proliferation in the mixed leukocyte culture. PBMCs were plated at 1 × 10^5^ cells in 96-well tissue culture plates in RPMI-1640 (BioloT, St. Petersburg, Russia) with 10% of autoplasma in the absence or presence of different macrophage subtypes (PBMCs : macrophages 10 : 1). Cells were incubated for 4 days, followed by pulse-labeling with 1 *μ*Ci/well of [3H] thymidine for an additional 18 hr. The allostimulatory activity was expressed by a stimulation index calculated as PBMC proliferation in the presence of macrophages/spontaneous PBMC proliferative response. For evaluation of the phenotype, macrophages were stained with CD206 (BD Biosciences, USA).

### 5.5. Evaluation of SH-SY5Y Cells Proliferation, Cell Yield, and Viability

Cells were cultured for 3 and 7 days in 96-well plates (TPP) at 1 × 10^4^ cells per well for the radioisotope method and at 2.5 × 10^4^ per well for the WST test. For assessing cell viability and yield, cells were cultured in 6-well plates at 4 × 10^6^ cells per well. SH-SY5Y cell proliferation was assessed after 5 days by adding [3H] thymidine (1 *µ*Ci/well) for 18 hr. Cells were then harvested, and the thymidine incorporation was measured in a liquid scintillation counter SL-30 (Intertechnic, France). Also, proliferation was evaluated by the WST test (Merck, Darmstadt, Germany) according to the manufacturer's instructions. The cell yield was assessed using a LUNA-II automated cell counter (Logos Biosystems, Annandale, VA, USA). Cell viability was assessed by flow cytometry using FACSCalibur and CellQuest software (BD Biosciences, San Jose, CA, USA), 7-aminoactinomycin-D (7-AAD) (Merck, Darmstadt, Germany) positive cells were determined to be nonviable.

### 5.6. Evaluation of SH-SY5Y Cell Differentiation

To assess SH-SY5Y cell differentiation, morphometric analysis was performed using the ImageJ program (National Institutes of Health (NIH), Bethesda, MD, USA) and the NeuronGrowth plugin [36, 37]. At least 100 cells in four different fields of view were analyzed, and three independent experiments were performed. The number of cells with neurites, the length of which exceeded the length of the cell body, and the average neurite length were used as morphological criteria of differentiation. Morphometric analysis was performed on days 3 and 7. For each cell, the length of the primary neurite, defined as a single neurite, was measured. For cells with more than one neurite, the length of the longest neurite was measured. RA (Sigma-Aldrich) at a concentration of 10 *µ*M was used as an inductor of differentiation (positive control).

### 5.7. Statistical Analysis

Statistical analysis was performed using STATISTICA 8.0 software (StatSoft Inc., Tulsa, OK, USA). The data is presented as a median and an interquartile range (Me (IQR, 25%–75% quartiles)). To reveal a significant difference between the compared values, the Mann–Whitney nonparametric *U*-test, the Wilcoxon signed-rank test, and the *t*-test were used. Differences were considered statistically significant at *p* < 0.05.

## Figures and Tables

**Table 1:** Effect of macrophage-conditioned media on the relative number of differentiated cells and the average neurite length in SH-SY5Y cultures under serum deprivation conditions.

Day	Parameter	SD	RA	+CM-M1	+CM-M2a	+CM-M2(LS)
3	Differentiated cells (%)	3.2 (0; 6.7)	35.2^†^ (32.3; 37.8)	6.9^‡^ (5.4; 8.3)	6.0^‡^ (5.2; 7.9)	14.9^†‡§^ (10.0; 19.8)
	Average neurite length (*μ*m)	25.1 (19.3; 34.3)	46.5^†^ (36.2; 60.7)	34.2^†‡^ (27.0; 46.6)	35.3^†‡^ (26.9; 46.9)	42.5^†‡§^ (32.4; 56.9)

7	Differentiated cells (%)	4.3 (1.5; 6.2)	38.2^†^ (35.7; 40.6)	5.0^‡^ (3.5; 5.6)	5.7^‡^ (4.6; 6.4)	12.9^†‡§^ (9.6; 15.4)
	Average neurite length (*μ*m)	27.5 (21.3; 38.0)	59.6^†^ (37.6; 75.6)	31.0^‡^ (24.2; 46.4)	34.5^‡^ (26.1; 44.0)	37.0^†‡^ (29.5; 48.7)

*Notes*. ^†^*p* < 0.05 compared to serum deprivation conditions (1% FBS); ^‡^*p* < 0.05 compared to RA (positive control of differentiation); ^§^*p* < 0.05 compared to CM-M1 and CM-M2a (the Mann–Whitney *U* test). CM, conditioned medium; RA, retinoic acid; SD, serum deprivation; FBS, fetal bovine serum. Data are presented as median (Q1; Q3 values). The results of three independent experiments are presented.

**Table 2:** Effect of macrophage CM on the relative number of differentiated cells and the average neurite length in SH-SY5Y cultures under serum deprivation and hypoxic conditions.

Day	Parameter	SD/HC	RA	+CM-M1	+CM-M2a	+CM-M2(LS)
3	Differentiated cells (%)	1.8 (0.0; 2.9)	31.6^†^ (28.7; 33.4)	6.8^†‡^ (6.2; 10.0)	8.3^†‡^ (4.6; 11.9)	17.7^†‡§^ (15.3; 20.0)
	Average neurite length (*μ*m)	25.2 (20.1; 35.5)	48.7^†^ (37.8; 63.0)	40.5^†^ (32.0; 50.7)	40.7^†^ (29.9; 52.5)	38.3^†^ (31.9; 57.1)

7	Differentiated cells (%)	0.0 (0.0; 0.0)	35.8^†^ (32.4; 35.8)	8.0^†‡^ (7.0; 8.8)	5.8^†‡^ (5.6; 9.5)	14.1^†‡§^ (13.3; 15.1)
	Average neurite length (*μ*m)	24.8 (20.7; 28.5)	59.3^†^ (44.9; 71.3)	41.5^†‡^ (32.4; 56.4)	45.5^†^ (36.1; 59.9)	55.5^†§^ (42.5; 64.8)

*Notes*. ^†^*p* < 0.05 compared to serum deprivation and hypoxic conditions (1% FBS, 100 *µ*M CoCl_2_); ^‡^*p* < 0.05 compared to RA (positive control of differentiation); ^§^*p* < 0.05 compared to CM-M1; *p* < 0.05 compared to CM-M2a (the Mann–Whitney *U* test). CM, conditioned medium; RA, retinoic acid; SD/HC, serum deprivation and hypoxic conditions; FBS, fetal bovine serum. Data are presented as median (Q1; Q3 values). The results of three independent experiments are presented.

## Data Availability

The authors confirm that the data supporting the findings of this study are available within the article.
